# Treatment of an intraarticular comminuted fracture of the base of the proximal phalanx in a ring finger using the Ichi-Fixator external fixator system: A case report

**DOI:** 10.1016/j.ijscr.2020.02.020

**Published:** 2020-02-13

**Authors:** Akira Hara, Minoru Yokoyama, Satoshi Ichihara, Yuichiro Maruyama

**Affiliations:** a Department of Orthopedic Surgery, Juntendo University Urayasu Hospital, 2-1-1 Tomioka, Urayasu, Chiba, 279-0021, Japan; b Department of Orthopedic Surgery, Tanaka Neurosurgical Hospital, Tokyo, Japan

**Keywords:** Metacarpophalangeal joint, External fixator, Intraarticular fracture, Comminuted fracture

## Abstract

**Introduction:**

Comminuted fractures involving the articular surface of the base of the proximal phalanx are relatively rare. We treated a patient with this type of fracture by open reduction and internal fixation with a locked-wire-type external fixator (Ichi-Fixator System).

**Presentation of case:**

A 45-year-old man was injured because his ring finger was kicked during a Futsal game. Radiographs and computed tomography revealed a comminuted intraarticular fracture of the proximal phalanx of this ring finger. We treated the fracture with open reduction and K-wires and external fixation. We removed the K-wire and external fixator 5 weeks postoperatively and initiated range of motion exercises. Five months postoperatively, his finger motion was fully recovered without restriction.

**Discussion:**

Comminuted intraarticular fractures of the base of the proximal phalanx are usually treated with plating. Complications such as interference with excursion of the central slip and lateral bands, extensor tendon rupture, and plate prominence have been reported in these fractures. In our patient, the Ichi-Fixator System was useful as a distraction apparatus for metacarpophalangeal joint fixation.

**Conclusion:**

A comminuted intra-articular fracture of the base of the proximal phalanx was treated successfully using the Ichi-Fixator system.

## Highlights

• Intra-articular comminuted fracture of MP joint was relatively rare.
• MP joint of ring finger was injured.
• Eventually range of motion of affected finger was fully recovered.
• Ichi-Fixator System was useful for comminuted intraarticular fracture of MP joint.

## 1 Introduction

Fractures of the lateral volar base of the proximal phalanx are common injuries and usually represent with collateral ligament avulsion injuries. In contrast, comminuted fractures involving the articular surface of the base of the proximal phalanx are relatively rare and usually represent as a volar base fracture with a central depression of the articular surface. Failure to reduce and secure the fracture leads to persistent subluxation, articular incongruity, and post-traumatic arthritis. These fractures are generally approached through a dorsal extensor-tendon-splitting incision [1] or volar A1 pulley approach to visualize the articular surface [2]. Most commonly, a significantly-sized palmar–ulnar or palmar–radial fragment exists, and fixation can be accomplished with a minicondylar plate or K-wires. We experienced a patient with a comminuted intraarticular fracture of the base of the proximal phalanx, which we treated by open reduction and internal fixation with a locked-wire-type external fixator (Ichi-Fixator System (IFS); Neo-medical, Saitama, Japan) [3,4]. The work has been reported in line with the SCARE criteria [5].

## 2 Case report

**Fig. 1:**
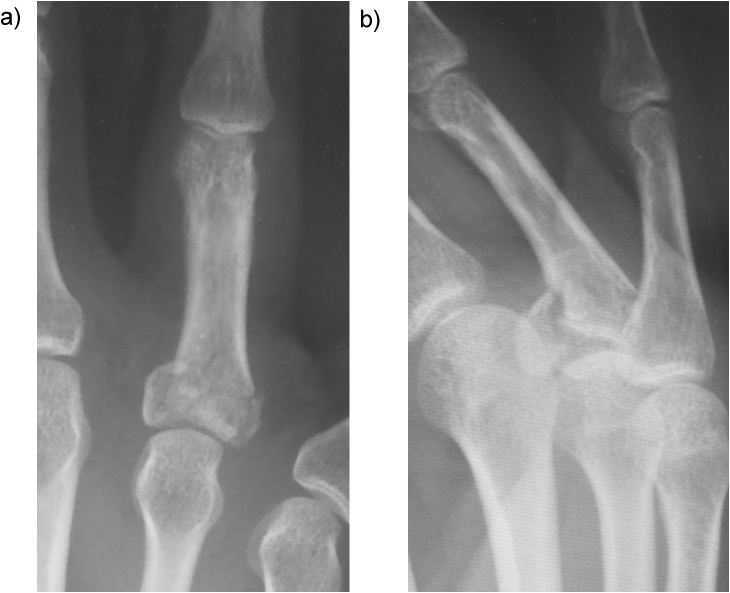
Preoperative X-ray showing a comminuted intraarticular fracture of the base of the proximal phalanx of the ring finger.

**Fig. 2:**
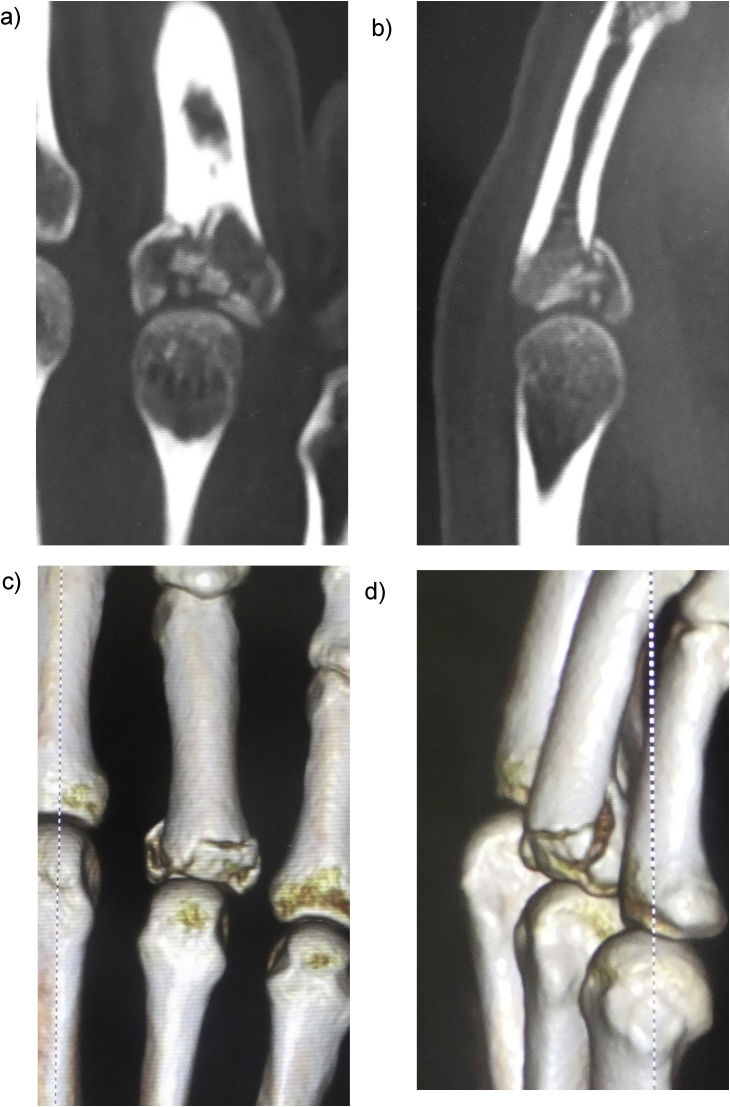
Plain computed tomographic and three-dimensional computed tomographic images showing a comminuted intraarticular fracture with central depression; A) coronal view, B) sagittal view, C) and D) three-dimensional computed tomographic images.

**Fig. 3:**
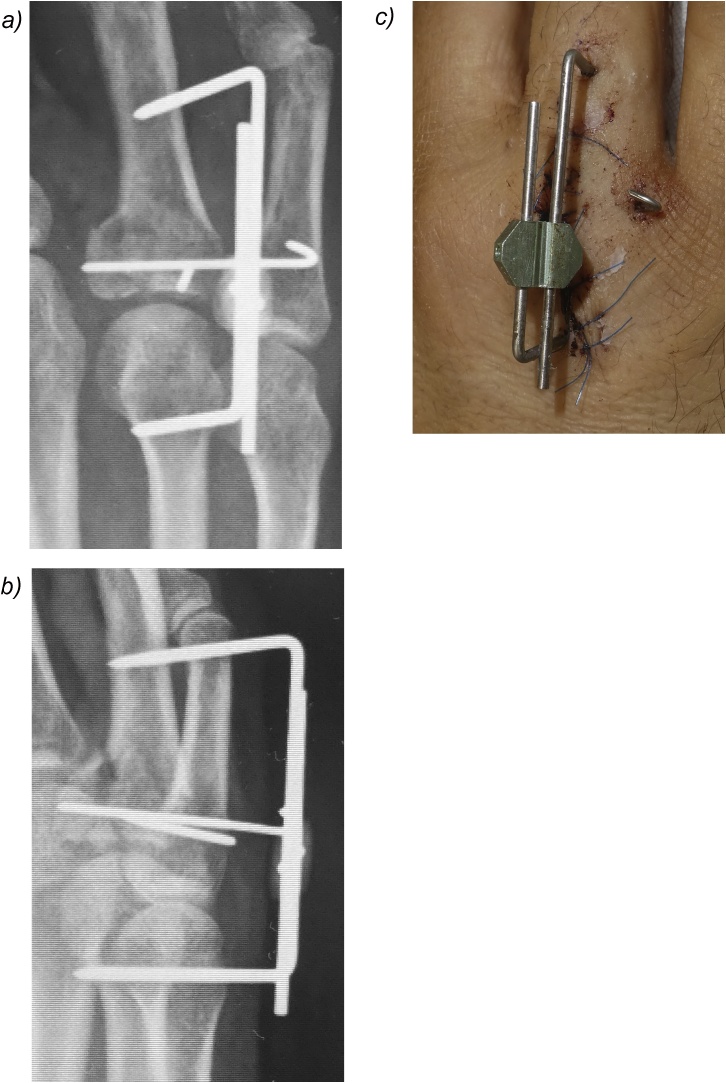
Postoperative X-ray A), B) and macroscopic findings C). The joint fragments were fixed with K-wires, and the Ichi-Fixator was applied to the ring finger metacarpophalangeal joint.

**Fig. 4:**
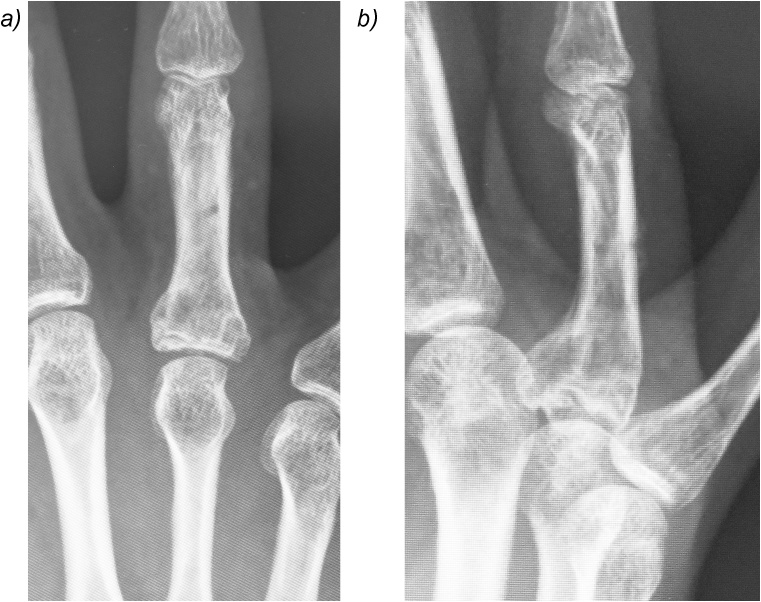
X-ray 5 months postoperatively showing the healed fracture.

**Fig. 5:**
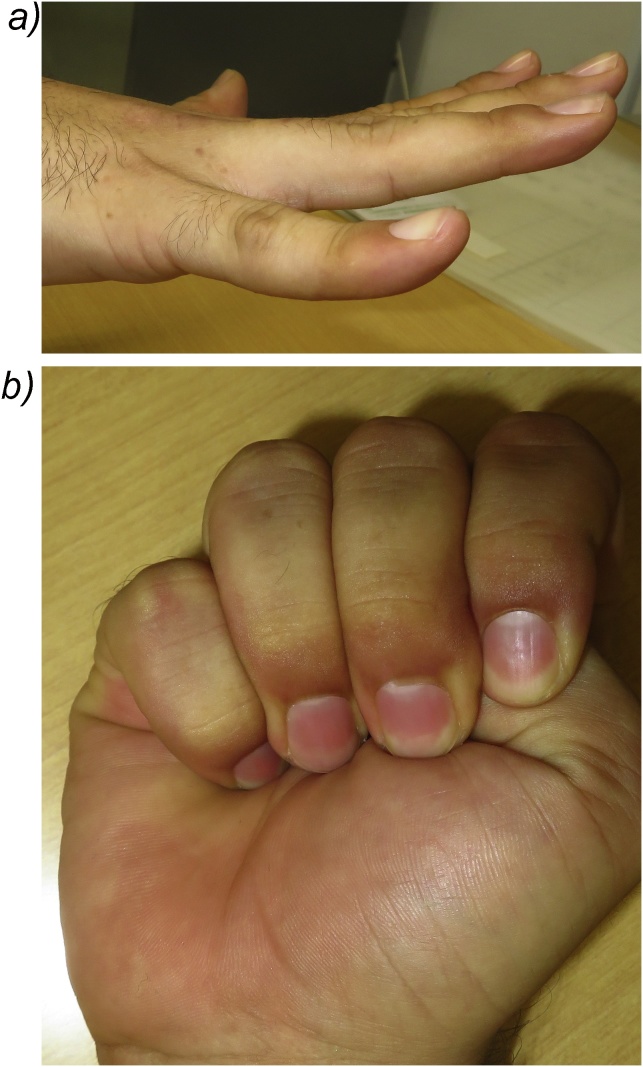
Total extension (a) and total flexion (b) of the patient's right hand.

A 45-year-old man presented to our hospital because of a ring finger injury. One day earlier, he played Futsal as a goalkeeper, when another player accidentally kicked the patient's ring finger while the patient was saving the ball. At presentation, his ring finger was swollen without a wound. Plain radiographs showed a comminuted intraarticular fracture of the base of his ring finger proximal phalanx (Fig. 1). Computed tomography revealed a comminuted fracture with articular depression of a fragment of the proximal phalangeal base (Fig. 2). We explained that closed reduction and conservative treatment would be failed. The patient chose operative treatment. Surgery was performed 10 days after his first visit. We approached the fracture site via dorsal extensor-tendon-splitting. The extensor mechanism was opened longitudinally, the dorsal capsule transversely, and the articular fracture visualized. The depressed central fragment was elevated and restored to the joint surface. We inserted two K-wires subchondrally in the dorsopalmar direction to reduce the joint fragments and sustain the joint surface in situ, which provided anatomical joint restoration. We applied the external fixator (IFS) to the proximal phalanx and metacarpal bone of the patient's ring finger as a distraction fixator to temporarily fix the fracture (Fig. 3). The external fixator and K-wires were removed 5 weeks after the operation, and active and passive range of motion exercises were encouraged. Three months after the operation, plain radiographs showed bone union without joint deformity (Fig. 4). Five months after the operation, the patient's finger motion was fully recovered without restriction (Fig. 5), and he returned to his previous work soon after the operation.

## 3 Discussion

Comminuted fractures involving the articular surface of the base of the proximal phalanx are relatively rare and are a challenge for hand surgeons because of the difficulty in achieving accurate reduction of the articular surface and obtaining secure fixation. These fractures usually present as a volar base fracture with a central depression of the articular surface, and are generally approached through a dorsal extensor-tendon-splitting incision to visualize the articular surface [1]. Fixation can be accomplished with a minicondylar plate or K-wires. Otherwise, the volar A1 pulley approach for open reduction and internal fixation with volar plating is used [2]. If the fracture is notably comminuted, skeletal traction or an external fixator should be considered. Although dorsal plating of the proximal phalanx provides rigid internal fixation that facilitates early range of motion exercises, complications and unsatisfactory results have been reported, namely, interference with excursion of the central slip and lateral bands, extensor tendon rupture, and plate prominence [6,7]. When volar plating was used, the potential complications were adhesion or bowstring of the flexor tendons, and plate prominence [2]. In our patient, because the joint surface was comminuted with joint depression, plate fixation was not an option. We chose open reduction of the joint fragments using the dorsal approach and fixation of the joint fragments with K-wires followed by applying the IFS. After anatomical reduction, the IFS provides relatively solid and temporary fixation as a distraction fixator. This apparatus is simple to apply, and has a low profile and minimal impact on the adjacent finger. Because the IFS can be used as a unilateral fixator, it can be used even for long or ring finger metacarpophalangeal joints where other external fixators such as the bilateral type or hinged external fixators are difficult to use.

## Sources of funding

We have no source.

## Ethical approval

It is retrospective case report and no ethical approval was required.

## Consent

Written informed consent was obtained from the patient for publication of this case report and accompanying images. A copy of the written consent is available for review by the Editor-in-Chief of this journal on request. Patient’s name, initials, or hospital numbers are not used in this manuscript.

## Author contribution

Akira Hara and Minoru Yokoyama conducted a literature search and drafted the manuscript.

Akira Hara and Minoru Yokoyama performed the operation.

Akira Hara and Satoshi Ichihara contributed during the patient management and participated in the design of the case report and coordination and helped draft the manuscript.

All authors read and approved the final manuscript.

Akira Hara wrote up.

Yuichiro Maruyama was consultant involved in management of patient, main guidance for write up.

## Registration of research studies

No.

## Guarantor

Akira Hara.

## Provenance and peer review

Editorially reviewed, not externally peer-reviewed.

## Declaration of Competing Interest

None of the authors have conflict of interest.

## References

[1] Hantings H. (1987). Unstable metacarpal and phalangeal fracture treatment with screws and plates. Clin. Orthop..

[2] Hattori Y., Doi K., Sakamoto S., Yamasaki H., Wahegaonkar A., Addosooki A. (2007). Volar plating for intra-articular fracture of the base of the proximal phalanx. J. Hand Surg..

[3] Ichihara S., Suzuki M., Hara A., Kudo T., Maruyama Y. (2018). New locked-wire-type external fixator (the Ichi-fixator) for fourth and fifth carpometacarpal joint dislocation. Case Rep. Orthop..

[4] Yamamoto Y., IchiSuzuki M., Hara A., Hidalgo D.J., Maruyama Y., Kaneko K. (2019). Treatment of finger phalangeal fractures using the Ichi-Fixator system: a prospective study of 12 cases. Hand Surg. Rehabil..

[5] Agha R.A., Borrelli M.R., Farwana R., Koshy K., Fowler A., Orgill D.P., For the SCARE Group (2018). The SCARE 2018 statement, For the SCARE Group: the SCARE 2018 statement: updating consensus Surgical Case Report (SCARE) guidelines. Int. J. Surg..

[6] Stern P.J., Wieser M.J., Reilly D.G. (1987). Complications of plate fixation in the hand skeleton. Clin. Orthop..

[7] Page S.M., Stern P.J. (1998). Complications and range of motion following plate fixation of metacarpal and phalangeal fractures. J. Hand Surg..

